# Supplementary material for: A machine-learning framework to characterize functional disease architectures and prioritize disease variants
Source: medRxiv. 2025 Oct 24:2025.10.23.25338598. Preprint. [Version 1] doi: 10.1101/2025.10.23.25338598 (PMC12633601; doi:10.1101/2025.10.23.25338598)
Supplement: Supplement 2 [file NIHPP2025.10.23.25338598v1-supplement-2.pdf]

medRxiv preprint doi: https://doi.org/10.1101/2025.10.23.25338598; this version posted October 24, 2025. The copyright holder for this preprint (which was not certified by peer review) is the author/funder, who has granted medRxiv a license to display the preprint in perpetuity.
It is made available under a CC-BY 4.0 International license .

Supplementary Figures

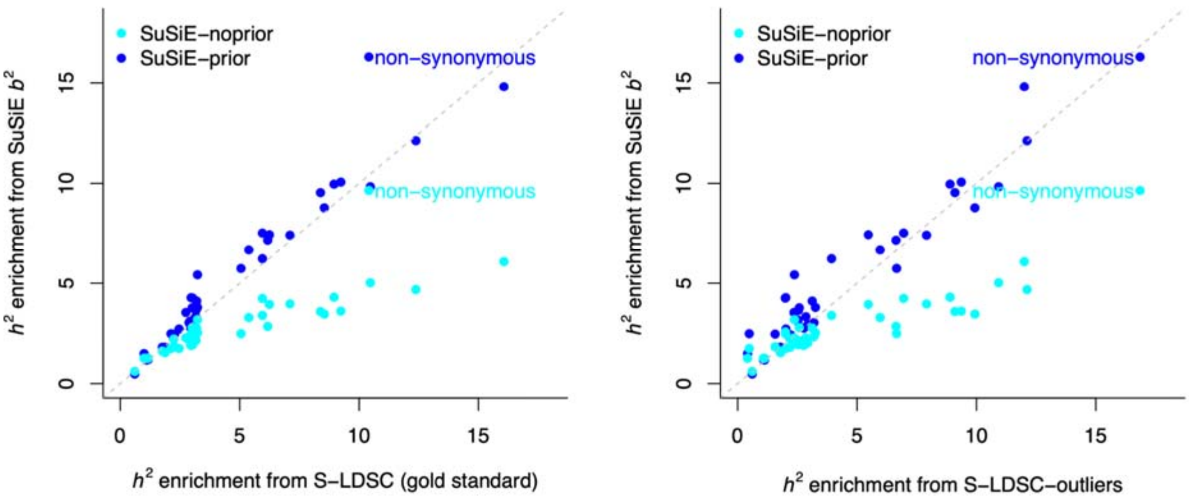

Figure S1. Comparing SuSiE b^2 estimates against S-LDSC for 15 UK Biobank traits. We report heritability (h^2) enrichment estimated using S-LDSC, SuSiE-noprior, and SuSiE-prior (priors obtained with PolyFun). The left panel is similar to **Fig. 3A** and shows default S-LDSC outputs, which exclude variants with high chi-square statistics. The right panel shows S-LDSC outputs obtained when including all the variants (S-LDSC-outliers); values were obtained by using the ldsc `--chisq-max 9999` option. For the non-synonymous annotation, S-LDSC-outliers yield higher enrichment than default S-LDSC, which suggests that default filtering underestimates h^2 enrichment for this annotation.

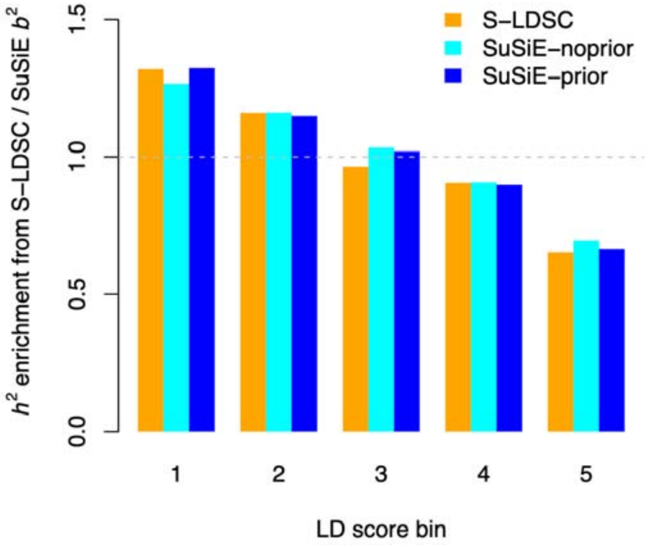

Figure S2. Evaluating SuSiE b^2 estimates on 15 UK Biobank traits. We report heritability enrichment estimated with S-LDSC (gold standard), SuSiE-noprior, and SuSiE-prior (PolyFun priors) across quintiles of LD scores. Numerical results and standard errors are reported in **Table S7**.

medRxiv preprint doi: https://doi.org/10.1101/2025.10.23.25338598; this version posted October 24, 2025. The copyright holder for this preprint (which was not certified by peer review) is the author/funder, who has granted medRxiv a license to display the preprint in perpetuity.
It is made available under a CC-BY 4.0 International license .

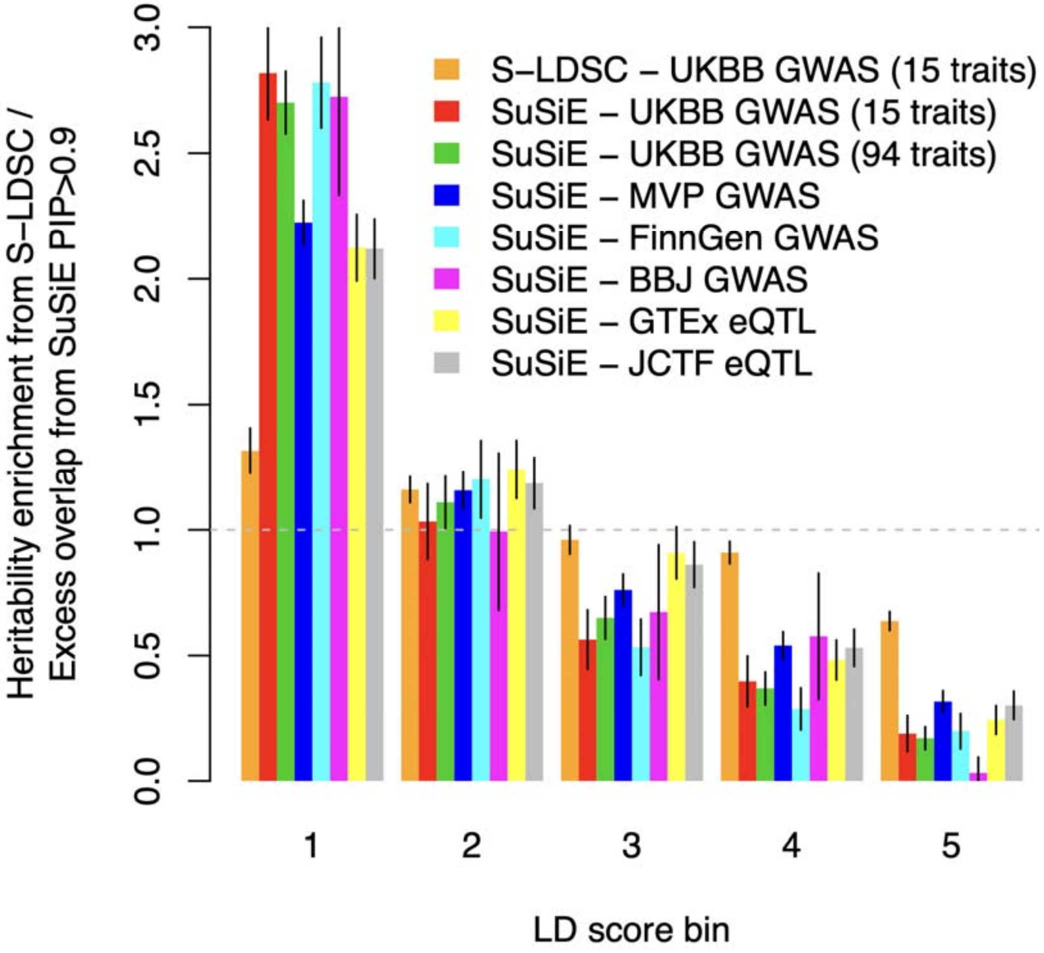

Figure S3. Variants that are confidently fine-mapped tend to have low LD scores. We report the excess overlap of variants that were confidently fine-mapped with SuSiE (PIP > 0.9) across seven datasets: the 15 independent UK Biobank traits used in this study (from ref. [11]), the 94 UK Biobank traits used in ref. [13], the 931 Million Veteran Program (MVP) GWAS used in refs. [13,19], FinnGen [20], Biobank Japan (BBJ) [32], eQTLs from GTEx [51,52], and the Japan COVID-19 Task Force (JCTF) [33]. S-LDSC estimates averaged across the 15 independent UK Biobank traits as a gold standard (here in orange) because we demonstrated that S-LDSC estimates for LD-related annotations are unbiased in simulations [3]. Variants fine-mapped with high confidence were disproportionately enriched in the lowest LD bin, which is consistent with their greater ease of fine-mapping.

medRxiv preprint doi: https://doi.org/10.1101/2025.10.23.25338598; this version posted October 24, 2025. The copyright holder for this preprint (which was not certified by peer review) is the author/funder, who has granted medRxiv a license to display the preprint in perpetuity.
It is made available under a CC-BY 4.0 International license .

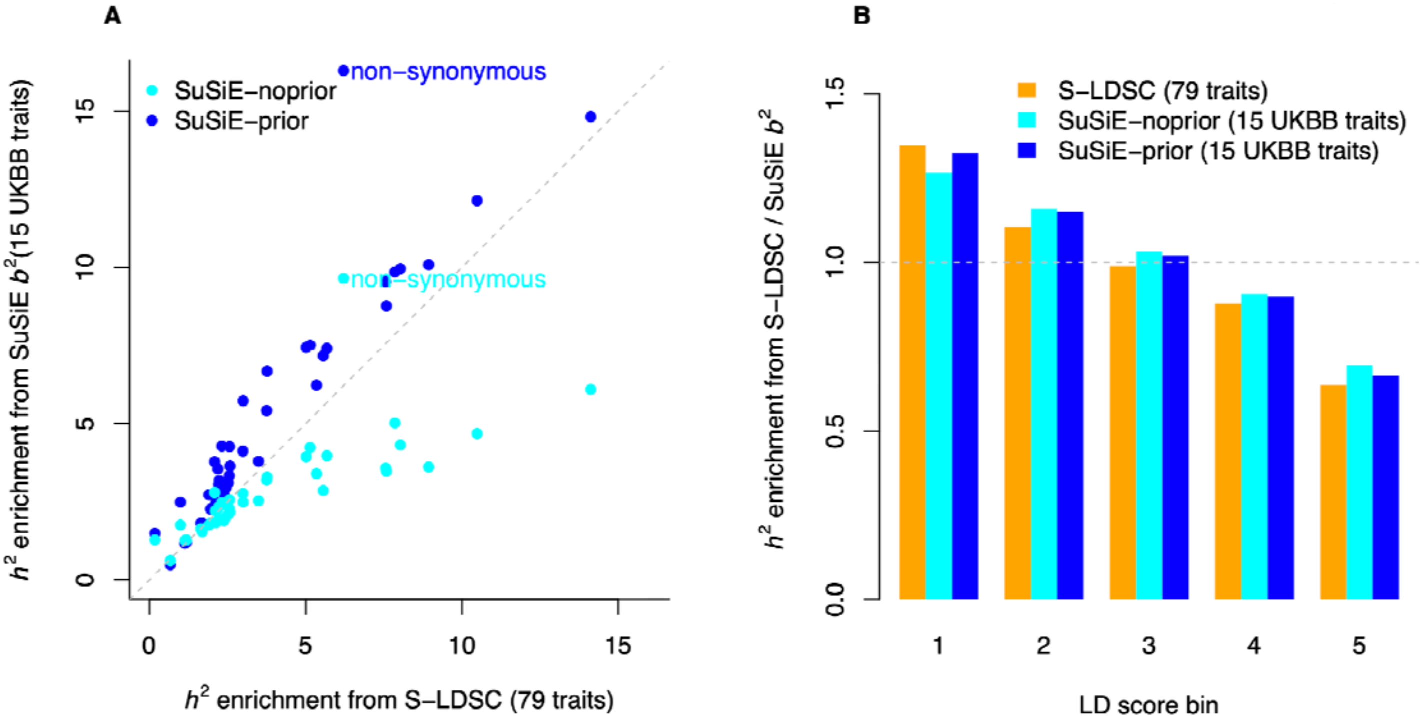

Figure S4. Comparing SuSiE *b*^{2} estimates on 15 UK Biobank traits against S-LDSC on 79 independent GWAS. (**A**) We report heritability enrichment for 40 main functional annotations, comparing SuSiE-noprior and SuSiE-prior (PolyFun priors) from 15 UK Biobank traits with S-LDSC estimates from 79 independent GWAS. (**B**) We report heritability enrichment within quintiles of LD scores. Results were similar to those in **Fig. 3**, indicating that SuSiE $_j^2$ estimates from 15 UK Biobank traits are representative of human diseases and complex trait genetic architecture.

medRxiv preprint doi: https://doi.org/10.1101/2025.10.23.25338598; this version posted October 24, 2025. The copyright holder for this preprint (which was not certified by peer review) is the author/funder, who has granted medRxiv a license to display the preprint in perpetuity.
It is made available under a CC-BY 4.0 International license .

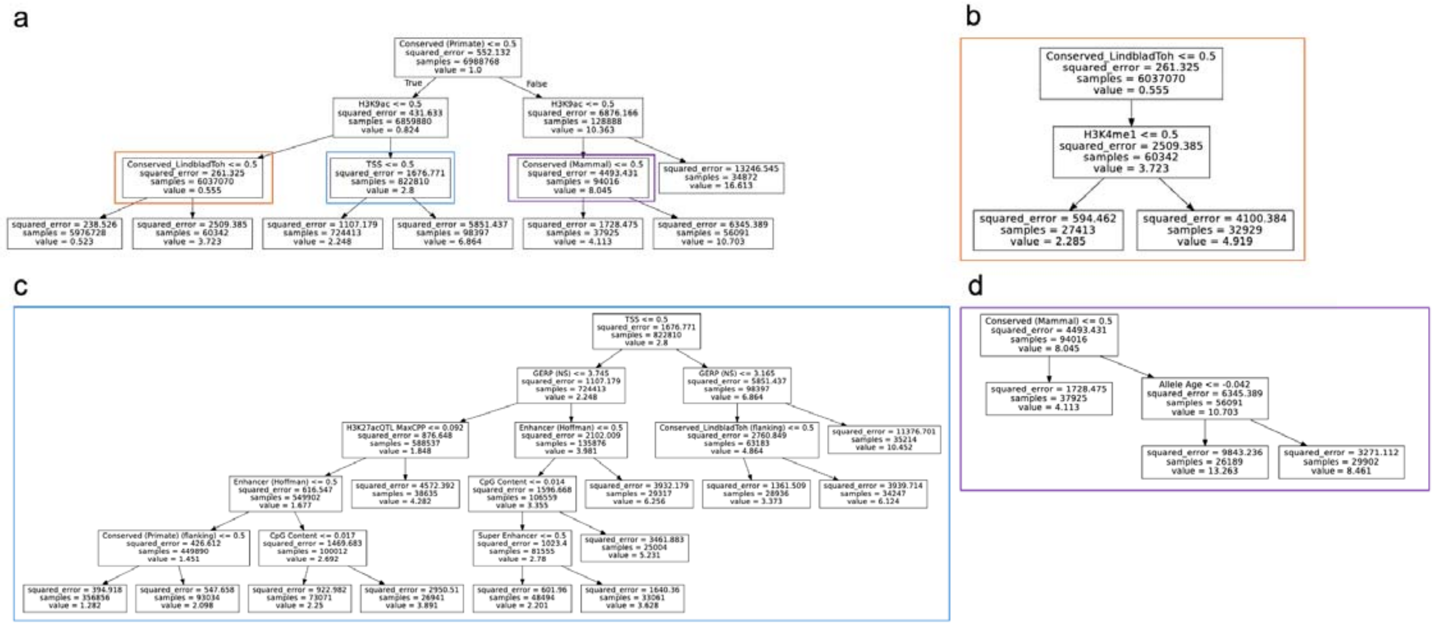

Figure S5. Decision tree using SuSiE-prior $_j^2$ on common variants. We report a decision tree with depth = 7 and minimum number of common SNPs per leaf = 25K obtained on normalized SuSiE-prior $_j^2$ estimates averaged across 15 UK Biobank traits. **(a)** Root of the tree (depth = 3). **(b–d)** Deeper branches**.** Each leaf reports the mean effect of the variant (value; normalized so the average effect of all common variants = 1), the number of SNPs in the leaf (samples), and the mean squared error (squared_error).

medRxiv preprint doi: https://doi.org/10.1101/2025.10.23.25338598; this version posted October 24, 2025. The copyright holder for this preprint
(which was not certified by peer review) is the author/funder, who has granted medRxiv a license to display the preprint in perpetuity.
It is made available under a CC-BY 4.0 International license .

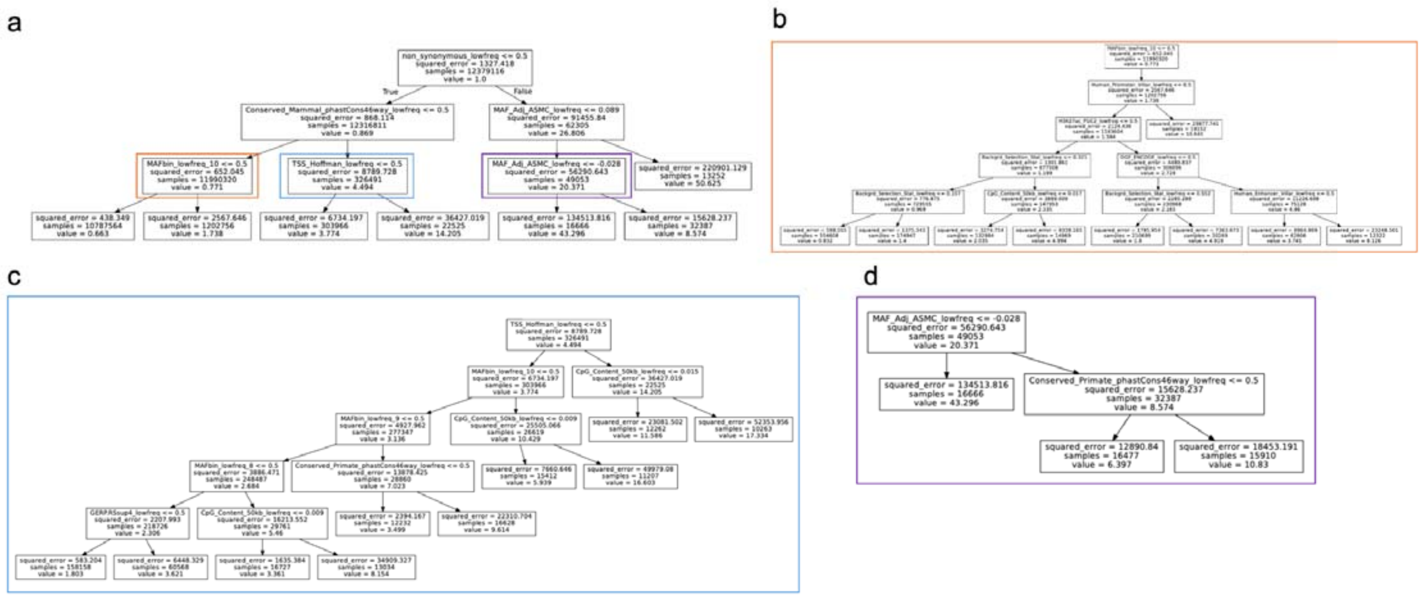

Figure S6. Decision tree using SuSiE-prior $_j^2$ on low-frequency variants. We report a decision tree with depth = 7 and minimum number of low-frequency SNPs per leaf = 10K obtained on normalized SuSiE-prior $_j^2$ estimates averaged across 15 UK Biobank traits. **(a)** Root of the tree (depth = 3). **(b–d)** Deeper branches**.** Each leaf reports the mean effect of the variant (value; normalized so the average effect of all low-frequency variants = 1), the number of SNPs in the leaf (samples), and the mean squared error (squared_error).

medRxiv preprint doi: https://doi.org/10.1101/2025.10.23.25338598; this version posted October 24, 2025. The copyright holder for this preprint (which was not certified by peer review) is the author/funder, who has granted medRxiv a license to display the preprint in perpetuity.
It is made available under a CC-BY 4.0 International license .

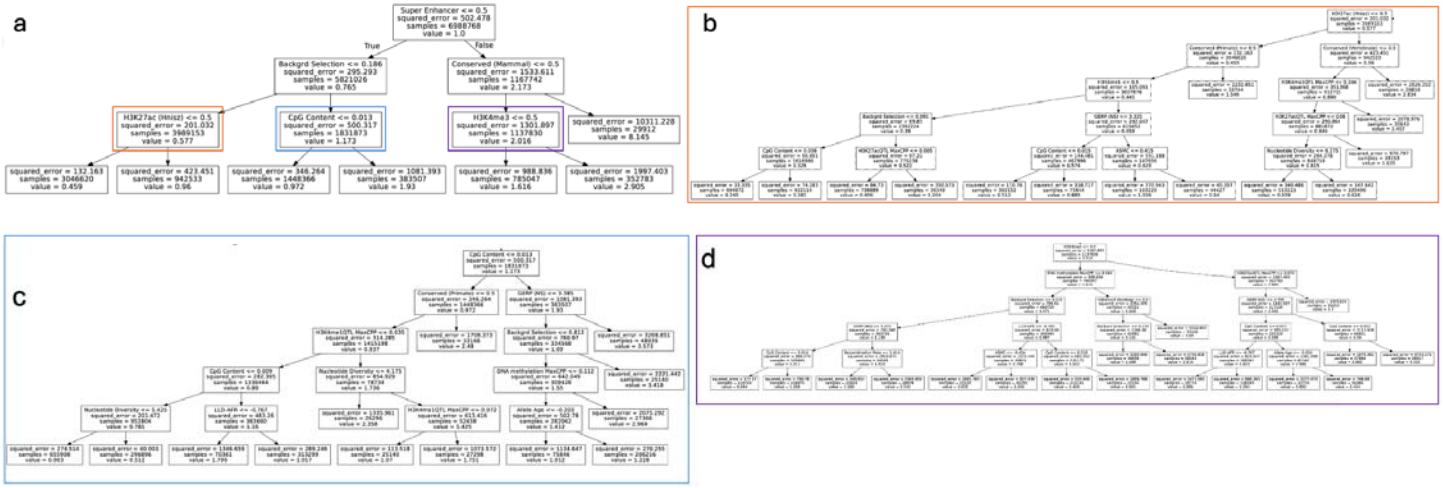

Figure S7. Decision tree using SuSiE-noprior $_j^2$ on common variants. We report a decision tree with depth = 7 and minimum number of common SNPs per leaf = 25K obtained on normalized SuSiE-noprior $_j^2$ estimates averaged across 15 UK Biobank traits. **(a)** Root of the tree (depth = 3). **(b–d)** Deeper branches**.** Each leaf reports the mean effect of the variant (value; normalized so the average effect of all common variants = 1), the number of SNPs in the leaf (samples), and the mean squared error (squared_error).

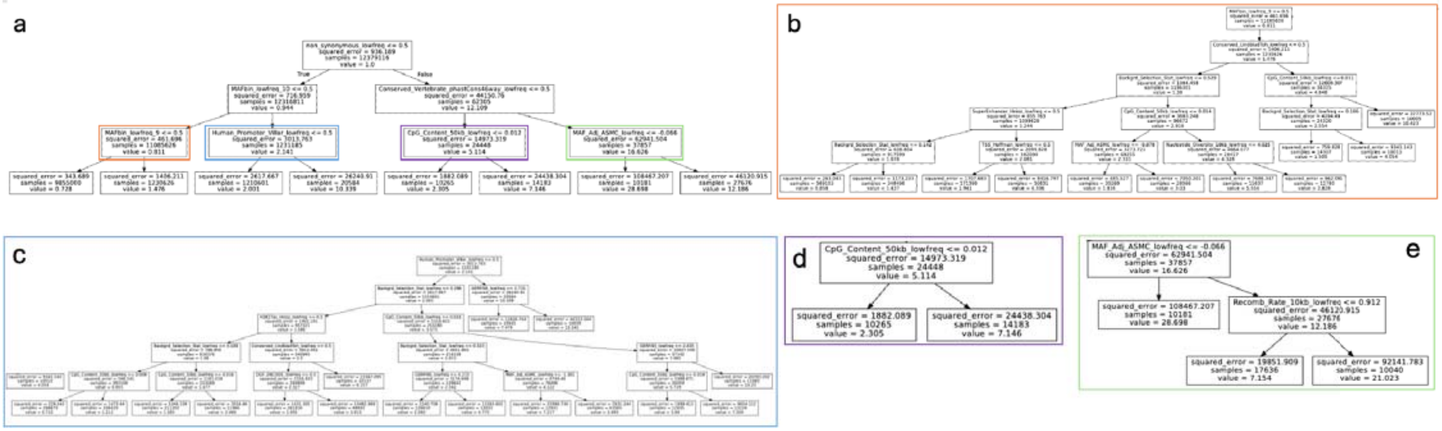

Figure S8. Decision tree using SuSiE-noprior $_j^2$ on low-frequency variants. We report a decision tree with depth = 7 and minimum number of low-frequency SNPs per leaf = 10K obtained on normalized SuSiE-noprior $_j^2$ estimates averaged across 15 UK Biobank traits. **(a)** Root of the tree (depth = 3). **(b–d)** Deeper branches**.** Each leaf reports the mean effect of the variant (value; normalized so the average effect of all low-frequency variants = 1), the number of SNPs in the leaf (samples), and the mean squared error (squared_error).

medRxiv preprint doi: https://doi.org/10.1101/2025.10.23.25338598; this version posted October 24, 2025. The copyright holder for this preprint (which was not certified by peer review) is the author/funder, who has granted medRxiv a license to display the preprint in perpetuity.
It is made available under a CC-BY 4.0 International license .

medRxiv preprint doi: https://doi.org/10.1101/2025.10.23.25338598; this version posted October 24, 2025. The copyright holder for this preprint
(which was not certified by peer review) is the author/funder, who has granted medRxiv a license to display the preprint in perpetuity.
It is made available under a CC-BY 4.0 International license .

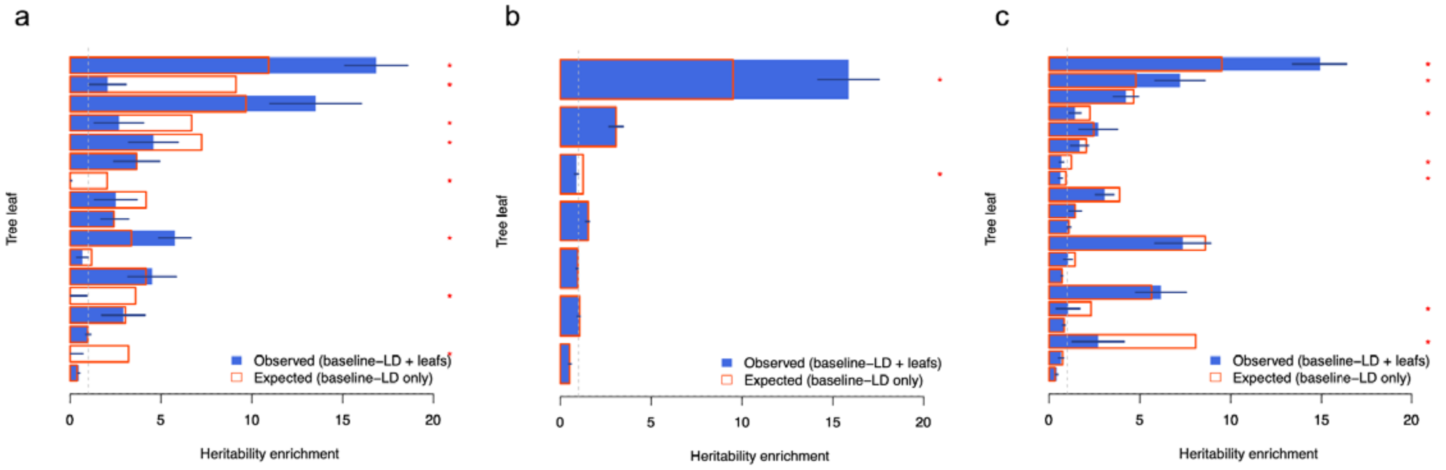

Figure S9. Heritability enrichment of common SNPs in leaves from decision trees. We report heritability enrichment of each leaf estimated with S-LDSC (blue) and compared to expectations under the baseline-LD model (red) using 79 independent European GWAS not overlapping the 15 UK Biobank traits. Error bars represent 95% confidence intervals, and asterisks indicate significant differences after Bonferroni correction. **(a)** We report enrichments for leaves constructed using SuSiE-prior $_j^2$ on decision tree with depth = 5; the leaf order (from bottom to top) is the same as plotted in **Fig. S5** (i.e., the top leaf corresponds to constrained in primates and in H3K9ac peaks). **(b)** We report enrichments for leaves constructed using SuSiE-noprior $_j^2$ on decision tree with depth = 3; the leaf order (from bottom to top) is the same as plotted in **Fig. S7**. **(c)** We report enrichments for leaves constructed using SuSiE-noprior $_j^2$ on a decision tree with depth = 5; the leaf order (from bottom to top) is the same as plotted in **Fig. S7**.

medRxiv preprint doi: https://doi.org/10.1101/2025.10.23.25338598; this version posted October 24, 2025. The copyright holder for this preprint
(which was not certified by peer review) is the author/funder, who has granted medRxiv a license to display the preprint in perpetuity.
It is made available under a CC-BY 4.0 International license .

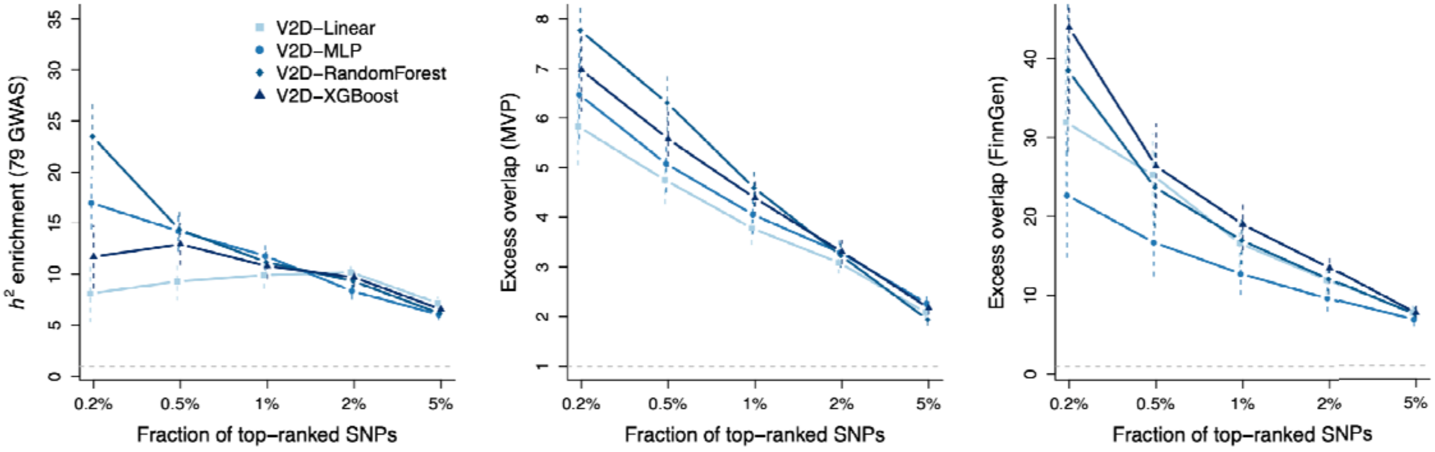

Figure S10. V2D scores obtained when using SuSiE-noprior $_j^2$. We report heritability enrichment across 79 independent GWAS and excess overlap with fine-mapped variants from MVP and FinnGen computed for V2D scores derived from linear, MLP, random forest, and XGBoost models. Error bars represent 95% confidence intervals.

medRxiv preprint doi: https://doi.org/10.1101/2025.10.23.25338598; this version posted October 24, 2025. The copyright holder for this preprint
(which was not certified by peer review) is the author/funder, who has granted medRxiv a license to display the preprint in perpetuity.
It is made available under a CC-BY 4.0 International license .

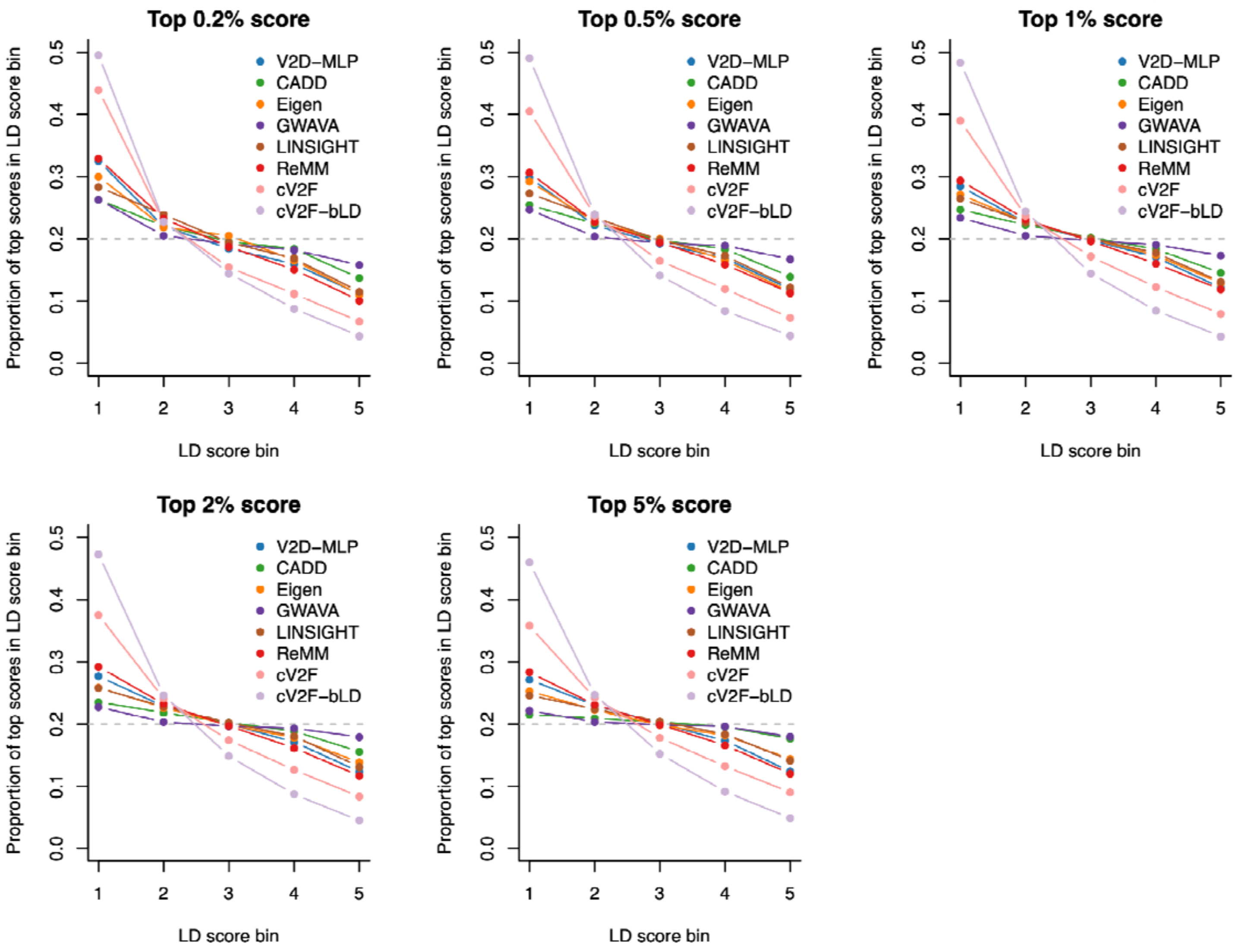

Figure S11. Proportion of top prioritization scores in LD score quintiles. We report the proportion of top-ranked variants within each LD score quintile for multiple prioritization scores. cV2F and cV2F-bLD are highly enriched (~2×) in low-LD variants but markedly depleted (~0.5×) in high-LD variants, thus reflecting their training on fine-mapped variants with high precision, which tend to occur in regions of low LD (see **Fig. S3**).

medRxiv preprint doi: https://doi.org/10.1101/2025.10.23.25338598; this version posted October 24, 2025. The copyright holder for this preprint (which was not certified by peer review) is the author/funder, who has granted medRxiv a license to display the preprint in perpetuity.
It is made available under a CC-BY 4.0 International license .

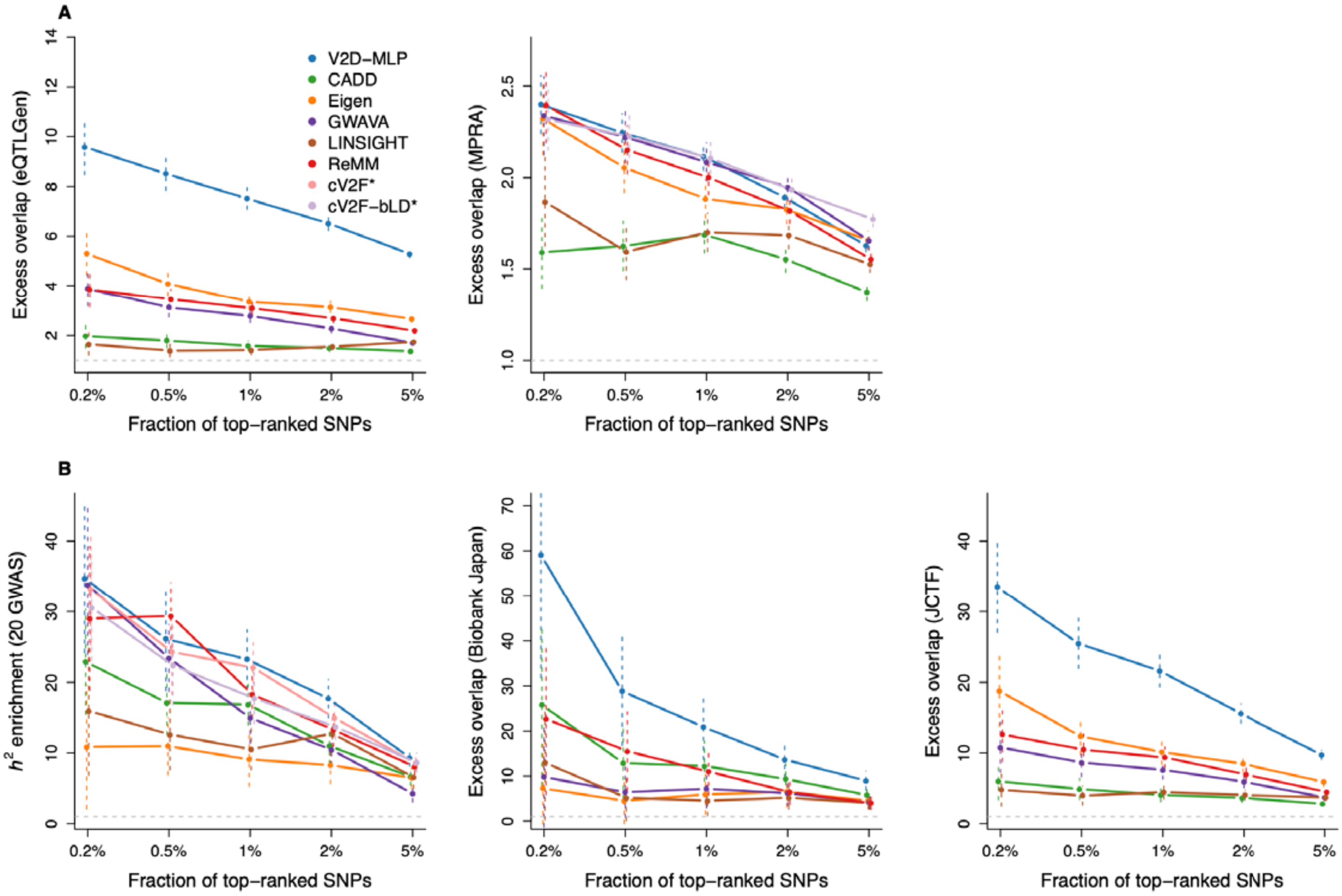

Figure S12. Benchmarking V2D scores across gene expression phenotypes and non-European ancestry datasets. **(A)** We report excess overlap on fine-mapped eQTLs from eQTLGen as well as 233K variants functionally tested using massively parallel reporter assay (MPRA) across five cell lines. **(B)** We report h^2 enrichment computed on 20 independent East-Asian GWAS and excess overlap computed on fine-mapped variants from 79 Biobank Japan GWAS and eQTLs from the Japan COVID-19 Task Force (JCTF). * cV2F and cV2F-bLD were not evaluated using fine-mapped datasets because cV2F scores are specifically trained on variants fine-mapped with high confidence and are inherently enriched toward SNPs with low LD versus other prioritization scores (**Figs. S3** and **S11**); cV2F was also not evaluated with the MPRA dataset because the same MPRA dataset is used as a feature of cV2F model.

medRxiv preprint doi: https://doi.org/10.1101/2025.10.23.25338598; this version posted October 24, 2025. The copyright holder for this preprint (which was not certified by peer review) is the author/funder, who has granted medRxiv a license to display the preprint in perpetuity.
It is made available under a CC-BY 4.0 International license .

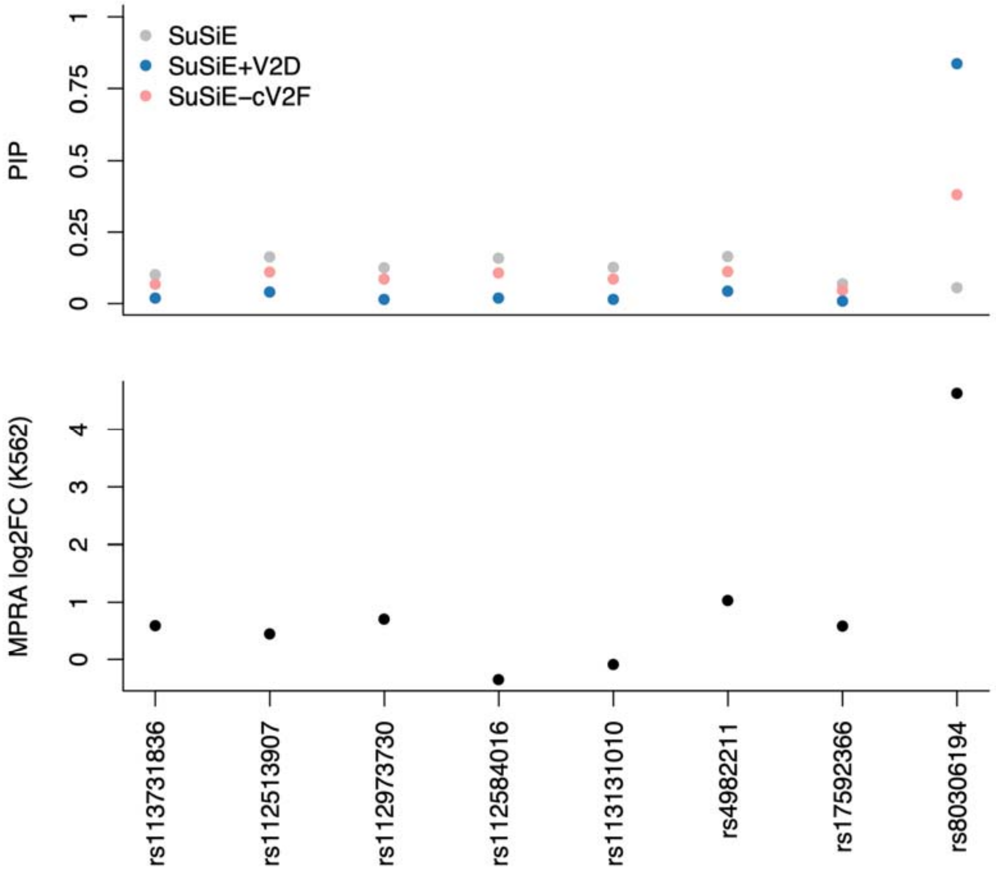

Figure S13. Fine-mapping and MPRA results at the *BAZ1A* locus of the red blood cell count GWAS. We report the posterior inclusion probabilities (PIPs) from SuSiE, SuSiE+V2D, and SuSiE+cV2F for the eight SNPs in the credible set (top). Corresponding MPRA log2 fold-change (log2FC) values in K562 cells are shown below (bottom).

medRxiv preprint doi: https://doi.org/10.1101/2025.10.23.25338598; this version posted October 24, 2025. The copyright holder for this preprint (which was not certified by peer review) is the author/funder, who has granted medRxiv a license to display the preprint in perpetuity.
It is made available under a CC-BY 4.0 International license .

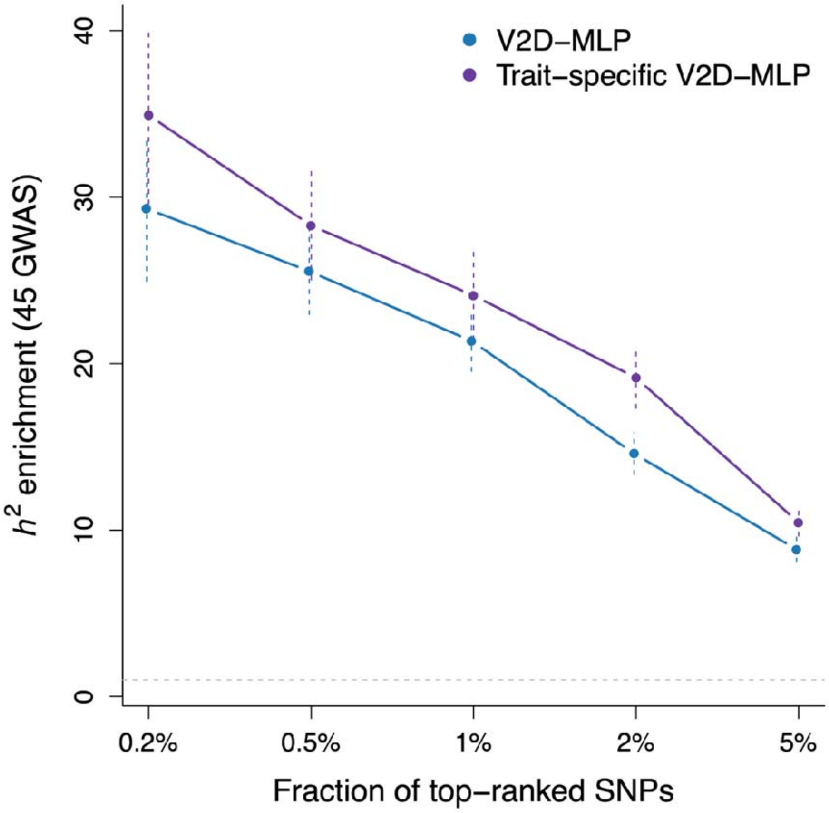

Figure S14. Heritability enrichments for V2D-MLP and trait-specific V2D-MLP scores. We report h^2 enrichment computed across 45 of 79 independent GWAS for which we were able to create trait-specific V2D-MLP scores. Error bars represent 95% confidence intervals. Enrichment obtained with trait-specific V2D-MLP scores were consistently higher than those obtained with V2D-MLP scores; differences were significant for the top 2% SNPs (P = 5.7 x 10^{-5}) and top 5% SNPs (P = 0.004); we note that the cell-type-specific annotations leveraged to compute trait-specific V2D-MLP scores captured a mean of 1.5% of common SNPs.

medRxiv preprint doi: https://doi.org/10.1101/2025.10.23.25338598; this version posted October 24, 2025. The copyright holder for this preprint
(which was not certified by peer review) is the author/funder, who has granted medRxiv a license to display the preprint in perpetuity.
It is made available under a CC-BY 4.0 International license .

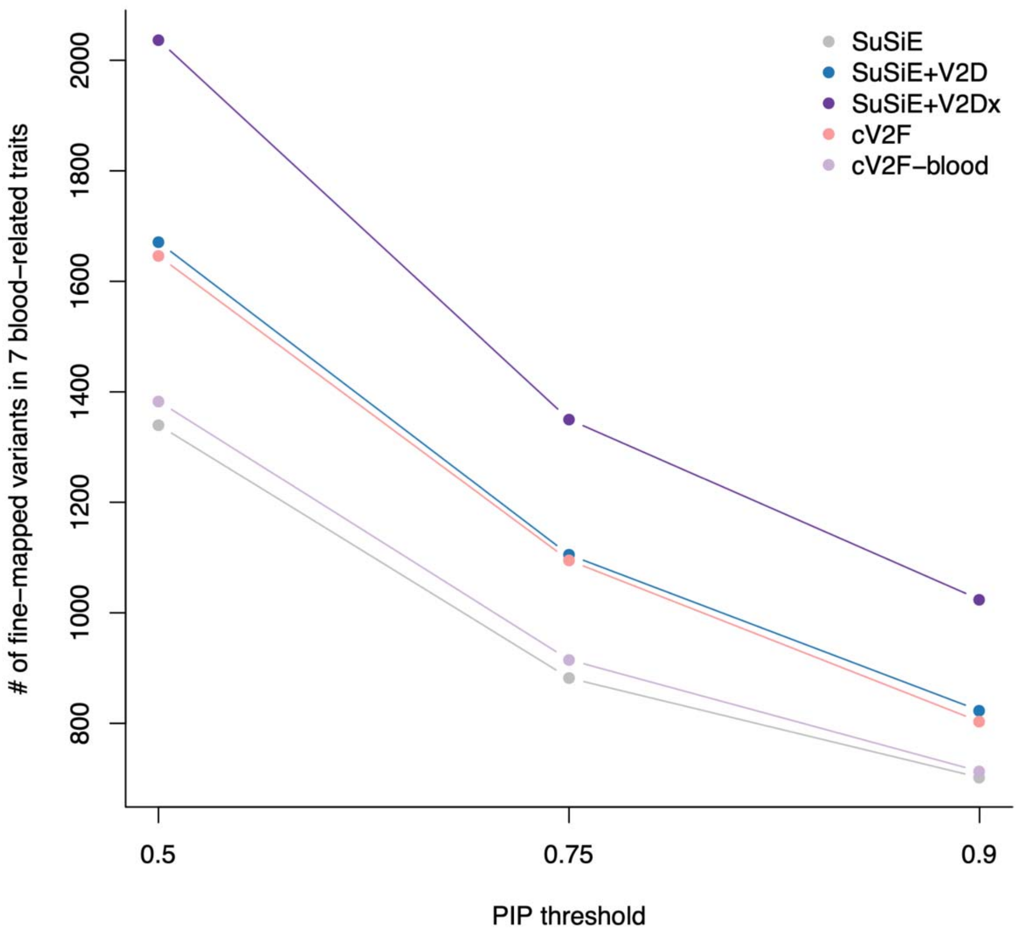

Figure S15. Leveraging trait-specific V2D scores to prioritize variants of blood-related traits. We report the number of fine-mapped SNPs exceeding PIP thresholds across seven UK Biobank blood-related traits.

medRxiv preprint doi: https://doi.org/10.1101/2025.10.23.25338598; this version posted October 24, 2025. The copyright holder for this preprint (which was not certified by peer review) is the author/funder, who has granted medRxiv a license to display the preprint in perpetuity.
It is made available under a CC-BY 4.0 International license .

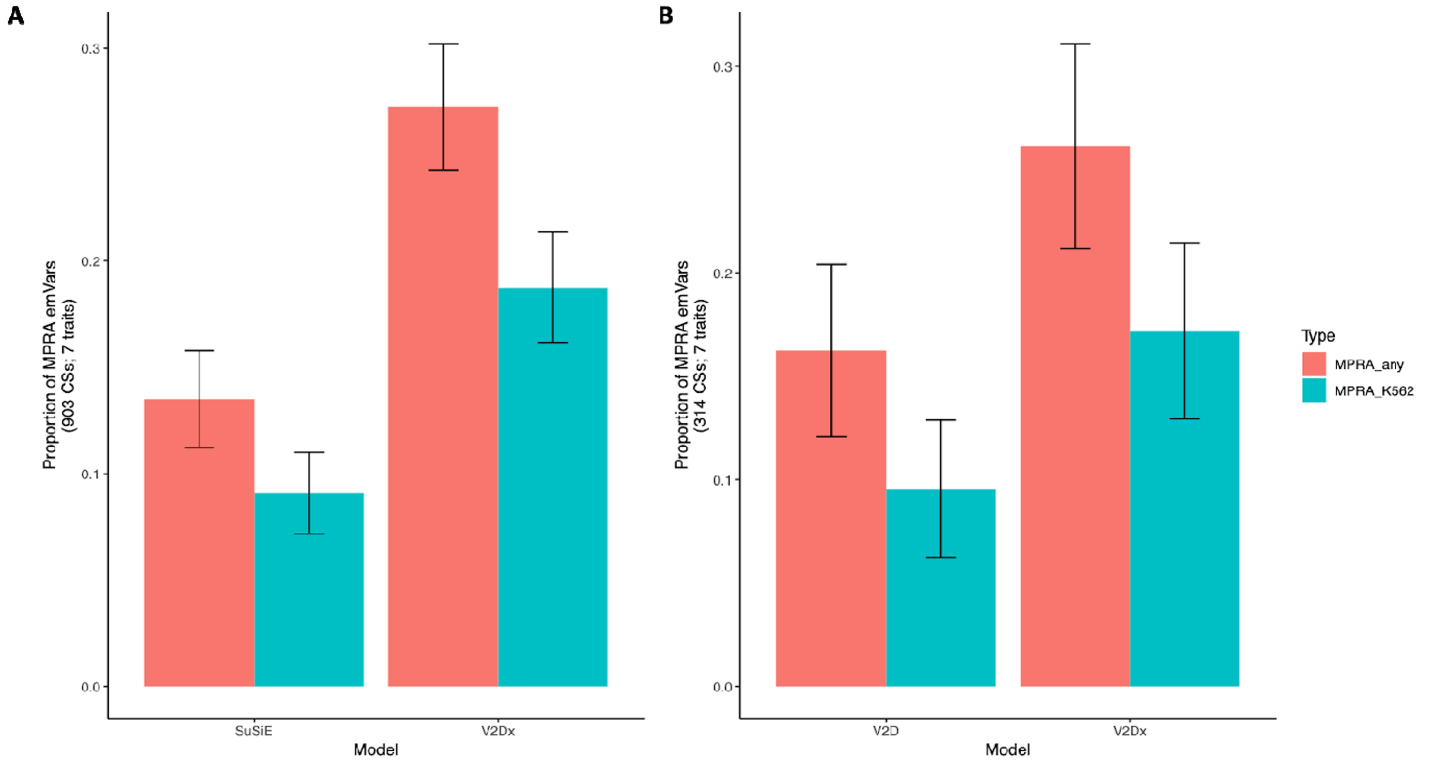

Figure S16. MPRA results for discordant credible sets in fine-mapping analyses of seven blood-related UK Biobank traits. We report the fraction of lead SNPs (variants with the highest PIP) that are expression-modifying variants (emVars) in credible sets with different lead SNPs from SuSiE and SuSiE+V2Dx (**A**) and SuSiE+V2D and SuSiE+V2Dx (**B**). Variants prioritized by SuSiE-V2Dx were significantly more likely to be emVars than variants prioritized by SuSiE (27.2 ± 1.5% vs. 13.5 ± 1.1% across 903 discordant CS; P = 6.1 x 10^{-13}) and SuSiE+V2D (26.1 ± 2.4% vs. 16.2 ± 2.0% across 314 discordant CS; P = 0.005). Also, variants prioritized by SuSiE-V2Dx were significantly more likely to be K562 emVars than variants prioritized by SuSiE (18.8 ± 1.3% vs. 13.5 ± 1.1% across 903 discordant CS; P = 8.5 x 10^{-9}) and SuSiE+V2D (17.2 ± 2.1% vs. 9.5 ± 1.6% across 314 discordant CS; P = 0.009).

medRxiv preprint doi: https://doi.org/10.1101/2025.10.23.25338598; this version posted October 24, 2025. The copyright holder for this preprint (which was not certified by peer review) is the author/funder, who has granted medRxiv a license to display the preprint in perpetuity.
It is made available under a CC-BY 4.0 International license .

Supplementary Tables caption

Table S1: List of the 15 UK Biobank independent GWAS. These 15 traits correspond to the 16 independent traits from Weissbrod et al. 2020 Nat Genet, from which we removed hair color because of low polygenicity.

Table S2: List of the 187 functional and evolutionary annotations from our baseline-LF models. We report the 187 annotations of the baseline-LF model. They include 96 annotations for common variants (identical annotations from the baseline-LD model) and 91 annotations for low-frequency variants. We highlight 40 main common annotations used in **Fig. 2** and **Fig. 3.**

Table S3: List of the 79 independent European GWAS not correlated with the 15 UK Biobank traits. We report the set of 79 independent European GWAS constructed by removing from the 107 independent traits from ref. [48] the traits that were genetically correlated to the 15 UK Biobank GWAS.

Table S4: Results of the simulations under the baseline-LD model. We report true and estimated heritability functional enrichments for 40 representative functional annotations of the baseline-LD model.

Table S5: Results of the simulations using an interactive effect between the coding and conserved annotations. We report true and estimated heritability functional enrichments obtained with SuSiE and with S-LDSC without and with the interaction modeled (S-LDSC and S-LDSC+interaction, respectively).

Table S6: Estimates of heritability enrichment estimated across 15 UK Biobank traits with S-LDSC, SuSiE-noprior, and SuSiE-prior. We report heritability functional enrichments for 40 representative functional annotations of the baseline-LD model. S-LDSC values were averaged across the 15 traits.

Table S7: Estimates of heritability enrichment across 15 UK Biobank traits estimated with S-LDSC, SuSiE-noprior, and SuSiE-prior. We report heritability functional enrichments for 5 LD score bins. S-LDSC values were averaged across the 15 traits.

Table S8: Mean squared error (MSE) of decision trees for common and low-frequency variants. We report MSEs computed using a LEOCO procedure on common variants and low-frequency variants. For interpretability, we multiplied b^2 by 10**7 and subtracted the MSE obtained by using the linear model (positive values mean that the decision tree does not provide a better fit than the linear model).

Table S9: Heritability explained by each leaf of the common variant tree. We report the common variant h^2 enrichment of each leaf estimated by S-LDSC and expected by S-LDSC with the baseline-LD model using 79 independent European GWAS not correlated with the 15 UK Biobank traits.

Table S10: Heritability explained by each leaf of the low-frequency variant tree. We report the low-frequency variant h^2 enrichment of each leaf estimated by S-LDSC and expected by S-LDSC with the baseline-

medRxiv preprint doi: https://doi.org/10.1101/2025.10.23.25338598; this version posted October 24, 2025. The copyright holder for this preprint (which was not certified by peer review) is the author/funder, who has granted medRxiv a license to display the preprint in perpetuity.
It is made available under a CC-BY 4.0 International license .

LF model using 23 independent UK Biobank traits with sufficient power to investigate low-frequency variant architecture.

Table S11: Benchmarking V2D scores across machine learning models. We report h^2 enrichment computed on 79 independent GWAS and excess overlap computed across common variants fine-mapped with high confidence in MVP and FinnGen for V2D scores obtained using different machine learning methods.

Table S12: MSE of machine learning models for common and low-frequency variants. We report MSE computed using a LEOCO procedure on common variants and low-frequency variants. For interpretability, we multiplied b^2 by 10**7 and subtract the MSE obtained by using the linear model (positive values mean that the model does not provide a better fit than the linear model).

Table S13: Benchmarking V2D scores across existing prioritization scores. We report heritability enrichment computed on 79 independent GWAS and excess overlap computed across common variants fine-mapped with high confidence in MVP and FinnGen for V2D scores obtained using existing prioritization scores.

Table S14: SuSiE+V2D results across 110 UK Biobank traits. We report SNPs with PIP > 0.50.

Table S15: SuSiE results across 110 UK Biobank traits. We report the number of SNPs with PIP greater than 0.5, 0.75 and 0.9.

Table S16. Expression-modifying variants (emVars) identified by SuSiE versus SuSiE+V2D.

Table S17. Expression-modifying variants (emVars) identified by SuSiE+cV2F-bLD versus SuSiE+V2D.
